# Treatment of Severe Hip Dysplasia with Leg Length Discrepancy Using Spherical Periacetabular Osteotomy

**DOI:** 10.1155/2022/3930806

**Published:** 2022-09-05

**Authors:** Yu Takeda, Tomokazu Fukui, Shigeo Fukunishi

**Affiliations:** Nishinomiya Kaisei Hospital, 4-1, Ohama-cho, Nishinomiya, Hyogo, Japan 662-0957

## Abstract

*Case*. A 20-year-old woman with severe subluxation of the hip displayed a leg length discrepancy of approximately 20 mm. The proposed treatment was a spherical periacetabular osteotomy (SPO) for joint preservation and postoperative leg length maintenance. SPO is a novel periacetabular osteotomy procedure. At her 3-year follow-up, the patient had functional, pain-free motion and high satisfaction. In this case, the SPO technique was able to achieve satisfactory clinical results without further exacerbating the leg length discrepancy after surgery.

## 1. Introduction

Periacetabular osteotomy (PAO) is the most popular surgical treatment of symptomatic developmental dysplasia of the hip (DDH) to preserve the hip joint. Curved periacetabular osteotomy (CPO) is also one of the several approaches for addressing hip dysplasia in adolescents and young adults [1]. However, several authors have reported that in patients with severe DDH who underwent CPO, a gap at the pubis osteotomy site due to overcorrection to improve acetabular coverage is associated with postoperative symptomatic superior pubis nonunion [2, 3]. On the other hand, the spherical periacetabular osteotomy (SPO) is a new minimally invasive PAO procedure that does not require a complete severance of the superior pubis and was first described by Kaneuji et al. in 2021 [4].

In this case report, we present a patient who underwent SPO for severe subluxation of the hip with leg length discrepancy with satisfactory clinical results.

The patient was informed and agreed to the data concerning her case being submitted for publication.

## 2. Case Presentation

The patient was a 20-year-old female whose gait suffered from mild hip pain during exercise and the leg length discrepancy in her lower left limb. The patient was aware that her leg length discrepancy was approximately 15 mm. When the passive motion test was administered, the patient expressed experiencing mild hip pain. The preoperative modified Harris hip score was 81 points.

**Figure 1:**
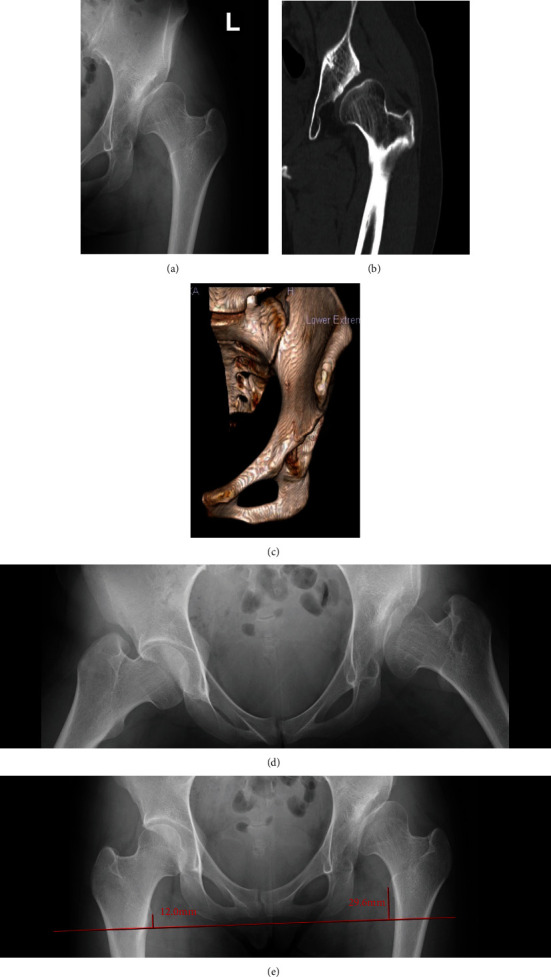
A 20-year-old woman with severe acetabular dysplasia. (a) Preoperative anteroposterior radiograph of the left hip joint, (b) preoperative CT coronal view, and (c) preoperative 3D-CT frontal view. Compatibility of the left hip joint was poor, and the femoral head of the superior acetabular coverage was insufficiently covered. (d) The abduction radiograph of the left hip shows good compatibility with the left hip joint. (e) The distance of leg length discrepancy from the center of the lesser trochanter to the bottom of the ischial tuberosity was 17.6 mm.

Preoperative plain anteroposterior (AP) simple radiographs of the hip joint showed a mild joint space narrowing with an acute medial angle of 52.3 degrees, a center-edge (CE) angle of 11.5 degrees, an acetabular roof obliquity (ARO) of 43.8 degrees, and acetabular head index (AHI) of 63.6% and a coxa plana. Preoperative CT images showed poor compatibility of the left hip joint and inadequate superior acetabular coverage (Figure 1). The pelvic radiograph of hip abduction showed good hip joint compatibility and maintenance of the joint space.

In the AP radiograph, the leg length discrepancy between the center of the lesser trochanter and the bottom of the ischial tuberosity was a distance of 17.6 mm. The patient's hip condition was diagnosed as severe subluxation of the hip (Hartofilakidis type II), and a periacetabular osteotomy was determined to be the best way to improve hip joint stability.

Initially, we proposed a modified Chiari pelvic osteotomy and a femoral varus osteotomy, but the patient did not agree to further postoperative shortening of the lower limbs because she was aware of the gait disturbance caused by the leg length discrepancy before surgery.

Therefore, a CPO was planned, but during preoperative planning, a review of the AP pelvic radiograph from the supine position showed a potential gap of more than 10 mm at the pubis osteotomy site. As a result, we altered the surgical plan from CPO to SPO to potentially reduce the chance of pubis nonunion.

The acetabular osteotomy plan for SPO was designed using a three-dimensional (3D) computed tomography (CT) templating system (OrthoMAP 3D, Stryker), and the osteotomy was performed following the SPO surgical procedure as described by Kaneuji et al. [4]. After correcting the bone fragment, *β*-TCP (tricalcium phosphate) was used to fill the gap between the host bone and the rotated bone fragment. The duration of the surgery was 263 minutes, and blood loss was approximately 4100 ml. Due to anemia caused by intraoperative bleeding, both an autologous (1140 ml) blood transfusion and a homologous (280 ml) blood transfusion were intravenously administered.

**Figure 2:**
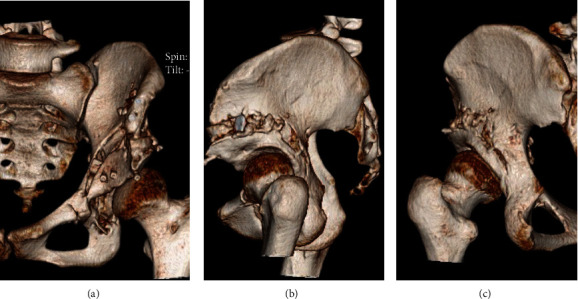
CT images taken one week after surgery: (a) frontal view, (b) sagittal view, and (c) posterior view. There was no fracture of the posterior column, and since the pubis was not completely osteotomized, there was no penetration of the osteotome into the joint, and the pubis was not completely cut. The pelvic ring has been preserved.

Postoperative CT images taken one week after surgery showed no posterior column fractures and no penetration of the osteotome into the joint. The rotated bone fragment was moved to the inferior-lateral position, and the pubis was not completely osteotomized (Figure 2).

**Figure 3:**
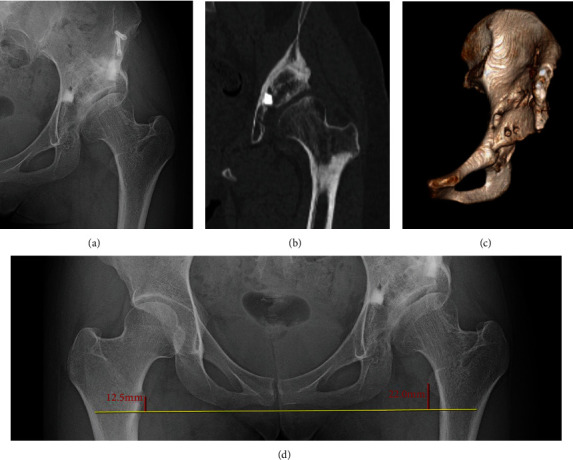
Anteroposterior (AP) radiograph and CT images taken three years after surgery showing the following: (a) AP radiograph of the left hip joint, (b) CT coronal view, and (c) 3D-CT frontal view. Although the superior acetabular coverage was still insufficient, the compatibility of the left hip joint was improved (d). When measured, the postsurgery leg length discrepancy was 9.5 mm.

In the postoperative AP radiograph of the hip taken three years after surgery, the radiographic parameter was a sharp angle of 44.9 degrees, CE angle of 17.6 degrees, ARO of 31.5 degrees, and AHI of 79.5%, and the leg length discrepancy was observed to be 9.5 mm. CT images from the patient's three-year follow-up also showed that the superior acetabular femoral head coverage and joint compatibility had improved. The rotated bone fragment and *β*-TCP recognized better union and remodeling (Figure 3). Range of motion and isometric exercise of the hip began after the patient was non-weight-bearing for one week. After four weeks, she was instructed to walk with touch down, and after sufficient adherence was observed, she gradually increased to full weight-bearing at the ten-week mark. Eleven weeks after surgery, the patient was discharged from the hospital with a T-cane.

At the most recent 3-year postoperative follow-up, the patient's modified Harris hip score was 95.7 points, and it was noted that she remained pain-free, engaged in regular physical activity, and gave birth naturally two years postsurgery.

## 3. Discussion

Various techniques of PAO have been reported as favorable procedures for preventing the development of osteoarthritis [5–8]. The ideal indication for PAO to correct acetabular dysplasia is in younger patients with early-stage osteoarthritis. However, PAO for severe acetabular dysplasia can sometimes be challenging as there is a high potential for postoperative complications due to a large amount of bone fragment transference. In this report, we present the case of a young woman who suffered from hip pain and whose daily routine was affected by an uneven gait due to severe hip subluxation and unequal leg length, which significantly improved after PAO.

Chiari pelvic osteotomy is a surgical procedure for severe hip subluxation in young adults, which has shown favorable clinical results [6]. On the other hand, complications have been reported with this procedure, including injury to the sciatic nerve, shortening of the affected extremity, a positive Trendelenburg gait, and interference with childbirth [8].

In order to improve joint congruency, an additional varus osteotomy would have been desirable, but further shortening of the lower extremity seemed inevitable in this case.

Rotational acetabular osteotomy (RAO), or CPO, is a popular approach for treating hip dysplasia in Japan, with good long-term clinical results [1, 6, 9]. CPO was reported to yield less soft tissue damage and early recovery abductor function via a more muscle-sparing approach than the transtrochanteric technique used by the modified Chiari osteotomy or RAO [1, 8, 10]. Thus, as it is a minimally invasive procedure, we have generally performed CPO for young patients diagnosed with DDH.

Both a Bernese periacetabular osteotomy and CPO require the cutting of the pubis to rotate the osteotomized bone fragment [4, 5].

In cases of CPO for severe hip dysplasia, significant movement of the acetabular fragment and overcorrection have been reported as postoperative complications as a result of superior pubic ramus nonunion [1, 2, 10].

Matsunaga et al. reported that a gap distance of more than 5.1 mm at the superior pubis osteotomy site is a risk factor for pubis nonunion [2]. Naito and Nakamura reported that the pubis ramus nonunion was asymptomatic in most patients and did not affect the clinical outcome [1]. However, nonunion rates of symptomatic pubis ramus were reported to range from 1% to 4% [2]. In our previous clinical practice, additional surgery with autologous bone grafting and plate fixation was performed in patients experiencing inguinal pain during exercise due to pubis nonunion after CPO.

First described in 2021, SPO is a novel periacetabular osteotomy procedure [4]. This periacetabular osteotomy is characterized as a procedure in which the complete osteotomy of the pubis is not required and can be performed using a minimally invasive, anterior approach similar to CPO. In this case, when considering CPO, the preoperative plan showed a distance of >10 mm at the pubis osteotomy site. Therefore, we decided to adopt an SPO as the treatment, considering the high possibility of pubis nonunion after surgery. Furthermore, SPO provides protection of inferior acetabular cortex continuity and sustained pelvic ring stability, which is expected to achieve a high rate of union [4].

In addition, postoperative leg length changes in SPO have been reported to range in various degrees (-6.23 mm to 6.82 mm) [11], and it is difficult to estimate the change in leg length following surgery. In this case, the patient had a leg length discrepancy of 17 mm caused by DDH, which greatly affected her gait. Therefore, it was necessary to prevent further leg shortening after surgery. Although Kaneuji et al. did not mention the change in leg length after SPO [4], we presumed that the postoperative leg length might be maintained or lengthened because the rotated bone fragments would move the femoral head center position inferiorly and laterally.

In this case, there was a wide gap between the host bone and the rotated bone fragment due to considerable acetabular coverage correction and leg length achievement. Three years after surgery, the bone union was satisfactory, and the radiographic parameter had improved. The patient continued to show improved leg length discrepancy during the postoperative radiograph evaluation. Additionally, osteoarthritis changes in the radiograph had not progressed during the final follow-up period. The patient continued to progress favorably; her gait and hip pain have improved, and she had given natural childbirth within the three years postsurgery.

However, there were problems associated with the surgical technique presented in this case. An acetabular roof inclination remained, and the hip joint was not adequately medialized. Kaneuji et al. also reported difficulty with femoral medialization as a problem associated with SPO [4]. Although the congruency of the left hip joint had improved, inadequate medializing of the hip center may lead to concentrated stress and further wearing of the joint. Therefore, long-term observation is necessary for the future progression of osteoarthritis. A long surgical time and considerable blood loss were also among the more serious problems of this surgery. Although there were no postoperative complications such as artery or nerve damage or nonunion of the osteotomy site, a shorter operative time may have reduced the amount of intraoperative blood loss. The patient had a large bone fragment transference and required prolonged postoperative hospitalization to prevent correction loss due to early weight-bearing.

In conclusion, a patient with severe DDH and suffering from a leg length discrepancy was treated with a novel PAO. This osteotomy technique is expected to minimize the risk of the pubis nonunion and preserve postoperative leg length, which may have promising short-term results for treating similar patients.
